# Adsorption Property, Kinetic and Equilibrium Studies of Activated Carbon Fiber Prepared from Liquefied Wood by Zncl_2_ Activation

**DOI:** 10.3390/ma12091377

**Published:** 2019-04-28

**Authors:** Ruoke Ma, Xianxian Qin, Zhigao Liu, Yunlin Fu

**Affiliations:** College of Forestry, Guangxi University, Nanning 530004, China; maruoke@163.com (R.M.); liu_zhi_gao@163.com (X.Q.)

**Keywords:** adsorption property, kinetic, adsorption isotherm, activated carbon fiber, liquefied wood

## Abstract

Activated carbon fiber was prepared from liquefied wood by chemical activation with ZnCl_2_ (Z-LWACF) at different impregnation ratios, with a particular focus on its adsorption property, kinetic and isotherm. The characterization and properties of Z-LWACFs were investigated by nitrogen adsorption/desorption, X-ray photoelectron spectroscopy (XPS), methylene blue (MB) and iodine adsorption. Two activation process methods were employed to prepare Z-LWACF and contrasted with others fibers. The results showed that the Z-LWACF obtained by one-step ZnCl_2_ activation present higher yields and specific surface area than others fibers. Besides, the change of MB adsorption value at different impregnation ratios was consistent with pore structure distribution above 1.5 nm pore size, indicating that larger micropores (1.5 to 2 nm) and mesopores played a major role in the MB adsorption by Z-LWACF. The kinetics of MB adsorption process was found to follow the pseudo-second-order kinetic model and the adsorption rate was controlled by chemisorption. It was also found that MB adsroption by Z-LWACF belonged to monolayer adsorption and Z-LWACF was easy to adsorb MB.

## 1. Introduction

Presently, several environmental problems, including the greenhouse effect, water pollution and air pollution have attracted increasing attention from governments and researchers all over the world. Dyes, one of water pollutants, have been widely used in many industries [1]. Methylene blue (MB), a cationic dye commonly applied in the textile and petroleum industry [2], can cause substantial harmful effects including vomiting, diarrhea, tissue necrosis and quadriplegia on human beings [3]. Besides, high concentrations of textile wastewater will impede the penetration of light and greatly reduce photosynthetic activity of aquatic organisms in the water ecological environment [4]. Thus, economical and high effective ecological treatment of MB containing wastewater will become more and more indispensable.

Various methods such as adsorption, advanced oxidation, biosorption and membrane filtration have been extensively applied in the treatment of contaminated water [5]. From the perspective of the initial cost of wastewater treatment technology, adsorption is an effective method with simplicity and ease of operation. Activated carbon fiber (ACF) can be considered as fibrous activated carbon. Fiber is characterized by high strength performance and free deformation. In addition, it does not cause secondary pollution and can also be made into other forms such as activated carbon fiber felt, cloth, mesh and paper in the application process. Currently, ACF is widely used in air purification, gas purification, industrial waste water treatment and water purification because of its excellent adsorption performance. Thus, air purifiers, dehumidifiers, and purifiers have been industrialized, and these products have also become a necessity in our daily lives.

Many raw materials, including polyacrylonitrile fiber [6], phenolic resin [7], pitch fiber [8], viscose fiber [9] and others biomaterials can be used as a precursor to produce ACF. However, those traditional raw materials are mostly non-renewable resources. Consequently, more and more researchers have focused on finding renewable resources for the preparation of ACF. In previously studies, ACF have been successfully prepared from some bio-resources such as wood, hemp, jute, ramie and bamboo [10,11,12,13]. Generally, activated carbon fibers exhibit different porosities and pore structure characteristics under various activation methods and treatment conditions. The adsorption performance of ACF is closely related to the special structure and chemical composition on the surface, and scholars have done a lot of research on its adsorption properties [14]. When the molecular diameter of adsorbent was larger, the adsorption value of ACF was related to the specific surface area [15,16], abundant mesopore and oxygen content [17,18]. Otherwise, ACF have been successfully prepared from liquefied wood [19] by various activation methods. Huang et al. [20] investigated the adsorption property of wood-based ACF by different activation methods and found that the chemical/physical coupling activation could greatly generate more mesopores resulting in improvement of MB adsorption capacity. Li et al. [21] had prepared activated carbon fibers from liquefied wood (WACFs) by CO_2_ activation and found the iodine adsorption amount and yield rate of WACFs were 779.22 mg/g and 51.48% at 900 °C. Adsorbate substance can be used to characterize different pore size structures based on their particle size. Due to the large molecular linear size of MB, it is generally used to characterize the pore structure with a pore size greater than 1.5 nm on the surface of the adsorbent or to evaluate the decolorization ability of ACF. In addition, Iodine molecules are spherical molecules with a diameter of about 0.54 nm, and the area occupied by adsorption is about 0.39 nm^2^ or more. Therefore, iodine adsorption is usually used for the micropore characterization of porous materials.

In this work, the activation process technology of liquefied wood carbon fiber by one-step method and two-step method were studied and evaluated. The iodine and methylene blue adsorption value of activated carbon fibers prepared from liquefied wood by ZnCl_2_ activation (Z-LWACF) under various impregnation ratios were measured. The effect of activation conditions on the microporous adsorption performance and decolorization ability of Z-LWACF was investigated. Furthermore, the adsorption equilibrium, kinetics and adsorption isotherm of MB on Z-LWACF were determined and correlated with isotherm equations.

## 2. Materials and Methods

### 2.1. Materials

*Chinese fir* (Cunninghamia lanceolate), sourcing from Fujian, China, was used as a raw material. Phosphoric acid, phenol, hydrochloric acid, hexamethyleneteramine, formaldehyde, sodium thiosulfate, disodium hydrogen phosphate, monopotassium phosphate, copper sulfate pentahydrate and zinc chloride were purchased from Beijing Chemical Works (Beijing, China). Soluble starch was purchased from Xilong Chemical Co. Ltd. (Shantou, China). Potassium iodide and iodine was purchase from Guangdong Guanghua Sc-tech Co. Ltd. (Shantou, China). Methylene blue was purchase from Tianjin Jinke Fine Chemicals Co. Ltd. (Tianjin, China) All the chemicals were of reagent grade and used without further purification.

### 2.2. Preparation and Characterization of ACFs

**Figure 1:**
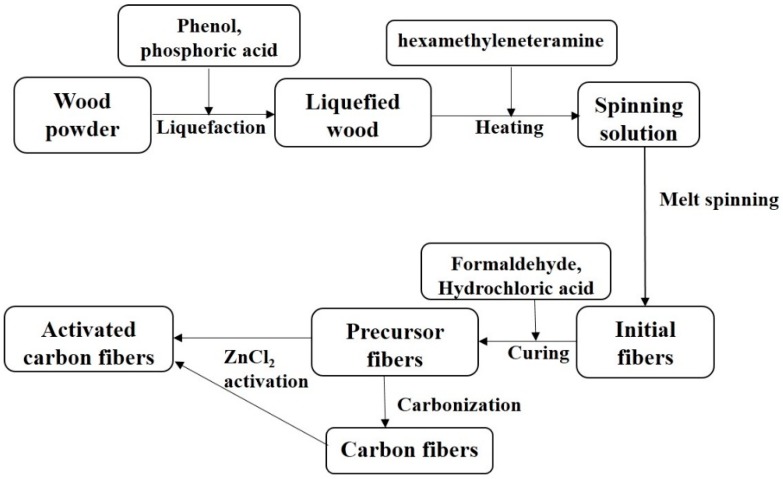
Preparation process diagram of activated carbon fiber by two methods.

Precursor fibers were prepared from *Chinese fir* through a series of process including liquefaction, melt-spinning and curing, according to our previous study by the authors [22]. In the impregnation of precursor fibers, 50 mL of ZnCl_2_ aqueous solution was brought into contact with about 2 g of the material. The amount of activating agent present in such a solution was that corresponding to a given ZnCl_2_: precursor fibers ratio (3~6 by weight). Then, ZnCl_2_-impregneated fibers were placed in a horizontal transparent tube furnace (Y02PB, Thermcraft Inc., Winston Salem, NC, USA) and heated from room temperature to 700 °C in a N_2_ atmosphere. The heated rate was 4 °C/min, and the holding time at 700 °C was 1 h. The produced sample were washed in 1 mol/L HCl, rinsed several times in distilled water until the pH of water became neutral. Finally, the fibers were dried in an oven at 103 ± 2 °C for 24 h to obtain the ACFs. The final yield of ACFs was determined as the weight ratio of ACFs to precursor fibers. The as-prepared ACFs were denoted as Z-LWACF-R, where R (3,4,5,6) represented the impregnation ratio. In addition, two activation methods were employed to prepare ACFs (Figure 1). These ACFs were denoted ZACF-700 and ZACF2-700. Meanwhile these two samples were compared with ACFs prepared by others activation methods in previous studies. The carbon yield (*Y*) is defined as the ratio of the weight of Z-LWACFs to that of the precursor fibers.

(1) $$Y=\frac{m_{z}}{m_{p}}\times 100\%$$

where *m*_p_ is the mass of the liquefied wood precursor fiber and *m*_z_ is the mass of Z-LWACF after activation, washing, and drying.

XPS analysis was performed using a spectrophotometer (Escalab 250Xi, Thermo Scientific, Waltham, MA, USA) with a monochromated AlKα X-ray source (hν = 1486.6 eV; 10 mA, 13 kV). The survey scans were collected from a binding energy ranging from 0 to 1350 eV. Pore structure parameters of those prepared ACFs were measured by N_2_ adsorption-desorption isotherms at −196 °C (Autosorb-iQ, Quantachrome, Boynton Beach, FL, USA). Before analysis, all the ACFs were degassed at 300 °C for 3 h. The specific surface area (*S*_BET_) of prepared ACFs was estimated by BET method. The total volume (*V*_total_) was estimated to be the liquid volumes of N_2_ at high relative pressure (*P*/*P*_0_ = 0.995). The micropore volume (*V*_micro_) and mesopore volume (*V*_meso_) were determined by t-plot analysis method and BJH method. The pore size distribution (PSD) of the prepared ACFs have been calculated by the density functional theory (DFT) method.

### 2.3. Adsorption Experiments

**Figure 2:**
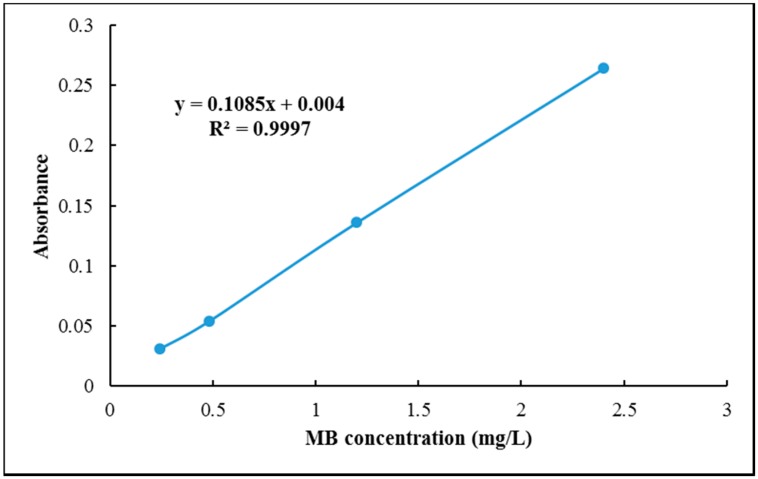
Standard curve of methylene blue.

The MB adsorption value of Z-LWACFs prepared under different activation conditions were determined according to the Chinese national standard GB/T 12496.10-1999 [23]. The prepared MB solution was diluted to a concentration of 0.24 mg/L, 0.48 mg/L, 1.2 mg/L and 2.4 mg/L with a buffer solution. Each aqueous solution was compared with copper sulfate standard filtrate and the absorbancy of various MB solution were measured as shown in Table 1 by UV spectrophotometer at a wavelength of 665 nm. The methylene blue standard curve is plotted as shown in Figure 2. The prepared sample was ground into powder and dried. About 0.100 g of Z-LWACFs was placed in a 100 mL erlenmeyer flask, and methylene blue reagent was added with the burette. These flasks were kept in isothermal shaker at 25 °C for 24 h. All the ACFs were filtered using a medium-speed qualitative filter paper which had a diameter of 12.5 cm in order to reduce the interference of samples with the measurement. After filtration, the filtrate was placed in a cuvette with 1 cm light path and absorbancy of aqueous sample was measured by using the UV spectrophotometer (Biowave II, WPA, Biochrom Ltd., Cambridge, UK) at a wavelength of 665 nm. The remaining concentrations of MB was calculated by using the standard curve. Furthermore, 25 mL of MB solution with initial concentrations of 120 mg/L, 240 mg/L, 360 mg/L, 480 mg/L, and 600 mg/L were prepared in advance and placed in erlenmeyer flasks. About 20 mg of Z-LWACF-6 samples was added to each flask and maintained under synthermal shaker at 150 rpm for 1 h, 2 h, 3 h, 4 h, 5 h, 12 h and 24 h. The concentration (*C*_t_) of MB was measured according to the method just described above, and finally the adsorption value (*Q*_t_) of Z-LWACF on different MB concentrations was calculated using the following equation:

(2) $$Q_{t}=\frac{(C_{0}-C_{t})V}{m}$$

where *C*_0_ is the initial MB concentration, *C*_t_ is remaining concentration at adsorption time, *V* is the volume of aqueous solution and *m* is the mass of Z-LWACF.

The iodine adsorption value of Z-LWACF prepared under different activation conditions was determined according to China national standard GB/T 12496.8-1999 [24]. About 0.5 g Z-LWACF was smashed firstly and added with 10 mL HCl into a 100 mL iodine volumetric flask in order to make the sample moist. These flasks were placed on the electric furnace and heated to boiling for 30 ± 2 s. When the temperature was cooled to room temperature, 50 mL iodine standard solution (0.1 mol/L) was added in the flask and kept in isothermal shaker for 15 min at 25 °C. After filtration, 10.0 mL of the filtrate was pipetted into a 250 mL iodine flask, and 100 mL water, was added. With sodium thiosulfate standard solution used in titration and 2 mL starch developing agent as indicator, iodine adsorption value (*A*) of Z-LWACF was calculated by the following equation:

(3) $$A=\frac{50\left(10C_{1}-1.2C_{2}V\right)127}{M}D$$

where *C*_1_ is the molarity of iodine standard solution, *C*_2_ is the molarity of sodium thiosulfate standard solution, *V* is the volume of sodium thiosulfate solution, *M* is the mass of Z-LWACF, *D* is the correction coefficient.

## 3. Results and Discussion

### 3.1. Comparisons with Activation Method

The yields and specific surface area of ZACF prepared from liquefied wood by one and two step methods and others ACFs were shown in Table 2. It can be observed that the yield and BET surface area of ZACF-700 were higher than those of ZACF2-700. Although the ZACF-700 and ZACF2-700 have been prepared under the same activation condition, ZACF2-700 was heated in two times leading to generate more by-products and the yield of ZACF2-700 was decreased. However, ZACF-700 has been directly activated by ZnCl_2_ after impregnation. The carbonization and activation have simultaneously occurred in the precursor fibers. Under low temperature, zinc chloride has a protective effect on the carbon body so that the precursor fibers can form a relatively stable condensed carbon structure and reduce the incidence of by-products. Then pores were produced and grown in the carbon fibers under the activation of ZnCl_2_, leading to a decrease of yield and an increase of specific surface area. Therefore, one step method would be more suitable for producing ACFs from liquefied wood by ZnCl_2_ activation. Furthermore, it is obvious from the data shown in Table 2 that the ACFs obtained by ZnCl_2_ activation present higher yields and BET surface area than those prepared by others methods [25,26,27,28,29]. This indicated that ACFs prepared from liquefied wood by ZnCl_2_ would show good adsorption properties.

### 3.2. XPS Analysis of Z-LWACFs

**Figure 3:**
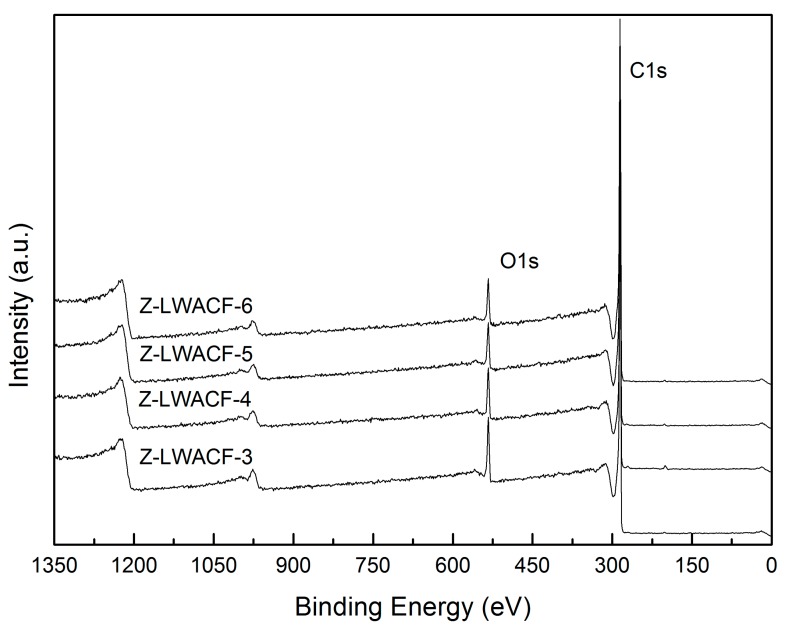
XPS survey scan spectra of Z-LWACFs prepared at different impregnation ratio.

**Figure 4:**
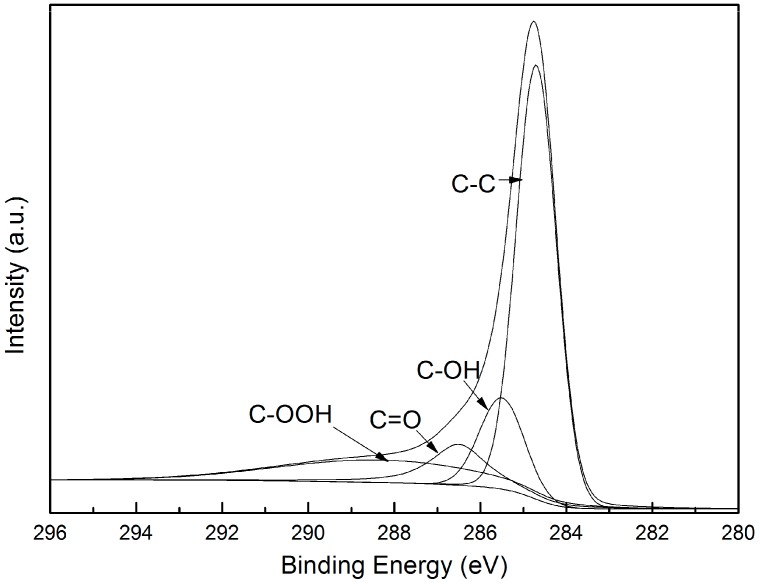
Peak fitting of C1s region of Z-LWACF-4.

The XPS survey scan spectra of ZnCl2-activated ACFs with various impregnation ratios are shown in Figure 3. It was apparent that the element C and O are dominant constituents in all samples. The species and relative content of carbon functional groups on the surface of Z-LWACFs can be obtained by fitting the peak of C1s in the X-ray photoelectron spectroscopy. Since the C1s peak spectrum curves of all the samples were very similar in shape, only one sample was used, which was shown in Figure 4. The background was deducted using Shirley’s method, and C1s peak of Z-LWACFs were fitted optimally by XPS PEAK41 software. As can be seen from the figure, the two sides of the main peak in the C1s region were not symmetrical, and there was a trailing phenomenon on one side. This was ascribed to the generation of oxygen-containing carbon group and graphite carbon on the surface of the ACF sample. As displayed in Figure 4, the C1s spectrum of ACFs were fitted into four peaks of established groups: graphite carbon (BE = 284.7 eV), C–OH (BE = 285.6 eV), –C=O (BE = 286.5 eV) and –COOH (BE = 288.3 eV) [30].

The curve-fitting results of C1s peak of Z-LWACFs prepared under different impregnation ratios are shown in Table 3. With the increase of impregnation ratio, the relative content of graphite carbon (C–C) decreased and the relative content of oxygen-containing groups increased gradually. This was attributed to the increase of zinc chloride concentration and enhancement of activation effect on carbon fiber. More graphite carbons have been oxidized to form oxygen-containing carbon functional groups on the surface, resulting in the decrease of graphite carbon content on the fiber surface. It was obvious that the relative content of C–OH was the highest among oxygen-containing functional groups. When the impregnation ratio increased from 3 to 5, the relative content of C–OH gradually increased, while the content of C=O and –COOH did not change much. This implied that the increase of zinc chloride could promote the oxidation reaction of the graphite carbon on the surface of the precursor and generate by-products like alcohol, ether or phenolic groups. In addition, while the impregnation ratio was 6, the relative contents of C=O and –COOH increased rapidly and the relative contents of C–O decreased from 24.25% to 22.20%, indicating that some C–O groups were further oxidized into ketone or acid groups under the action of activators. These functional groups would exert some effect on the surface performance of Z-LWACFs [31].

### 3.3. Effect of Impregnation Ratio and Pore Size on Adsorption of Methylene Blue

**Figure 5:**
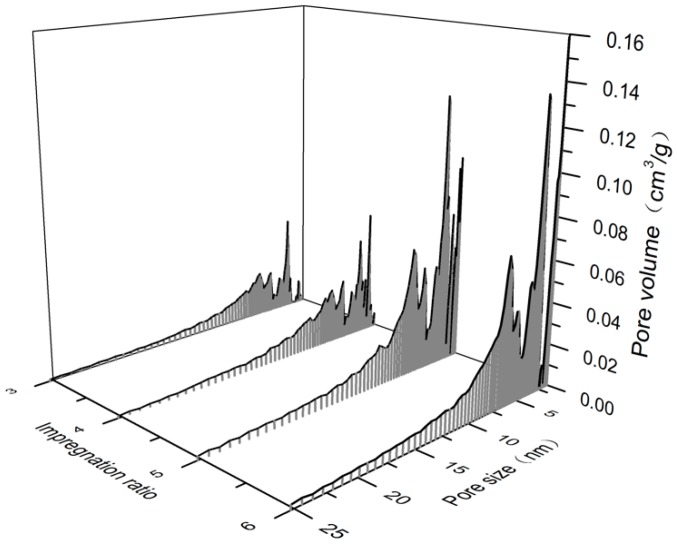
Pore size distributions of Z-LWACFs prepared at different impregnation ratios (1.5 nm to 25 nm).

Since the molecular linear size of methylene blue is large and the pore size of the adsorbent should be greater than 1.5 nm, MB is often used to characterize the decolorization ability of materials and the development degree of pore structure above 1.5 nm of porous materials. The MB adsorption value of ACFs prepared at different ZnCl_2_ impregnation ratios and the pore size distribution of pore structure in the range of 1.5 nm to 25 nm are shown in Table 4 and Figure 5. It can be seen from Table 5 that the amount of methylene blue adsorbed by the Z-LWACF sample gradually improved with the rising of the impregnation ratio, and reached a maximum value of 641 mg/g while the impregnation ratio was 6. As the impregnation ratio was increased from 3 to 6, the MB adsorption capacity of ZACF increased from 251 mg/g to 641 mg/g. The variation trend of MB adsorption amount of Z-LWACF with the impregnation ratio was basically consistent with the change trend of mesopore structure distribution. As displayed in the Figure 5, Z-LWACF had a narrow pore size distribution in the range of 3 and 4. However, with the increase of zinc chloride, the activation reaction was strengthened and the wall of smaller pores were further activated and eroded leading to enlarge the pore size. Therefore, the mesopore volume of Z-LWACF-5 and Z-LWACF-6 were greatly improved. The above results also indicated that the content of larger micropores (1.5 to 2 nm) and mesopores played a major role in the adsorption of MB by Z-LWACFs [32].

### 3.4. Effect of Impregnation Ratio and Pore Size on Adsorption of Iodine

**Figure 6:**
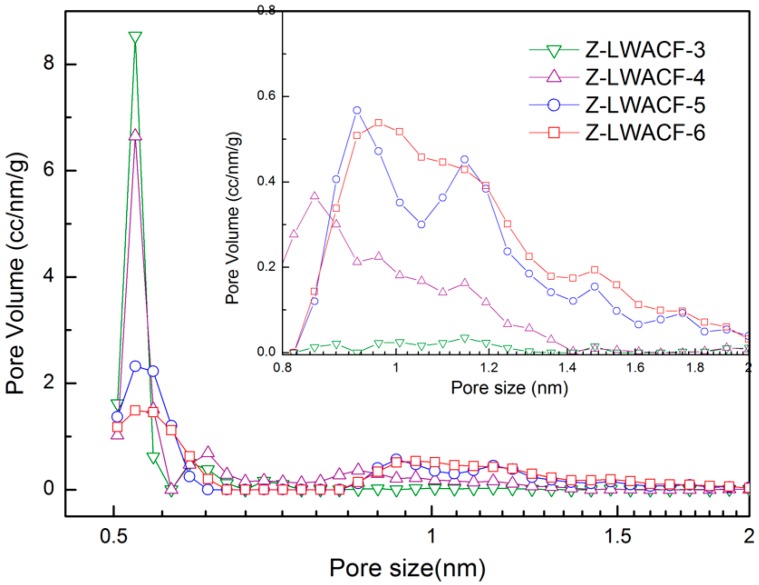
Micropore size distributions of Z-LWACFs prepared at different impregnation ratios.

Iodine adsorption mainly occurs on the surface of adsorbents with a pore radius of about 0.8 nm to 1.0 nm. It is usually used to reflect the adsorption capacity of activated carbon fibers for small molecules to a certain degree [33]. The iodine adsorption values and micropore size distributions of Z-LWACFs produced at different impregnation ratios are shown in Table 5 and Figure 6. It can be seen from Figure 6 that the pore volume of Z-LWACF decreases with the increase of the impregnation ratio at the pore size of 0.5 nm to 0.7 nm. In contrast, the pore volume of Z-LWACF was gradually increased at the pore size of 0.8 nm to 2 nm. This indicated that some micropores with small pore sizes were enlarged to the ones of larger size because of the more violent activation reaction as the impregnation ratio increased. Therefore, the iodine adsorption value of ZACF gradually increased with increasing impregnation ratio from 3 to 6, and when the impregnation ratio was between 5 and 6, the iodine adsorption value of Z-LWACF-5 and Z-LWACF-6 did not change much due to approximate pore volumes.

### 3.5. Effect of Contact Time and Initial Mb Concentration on Adsorption Equilibrium 

**Figure 7:**
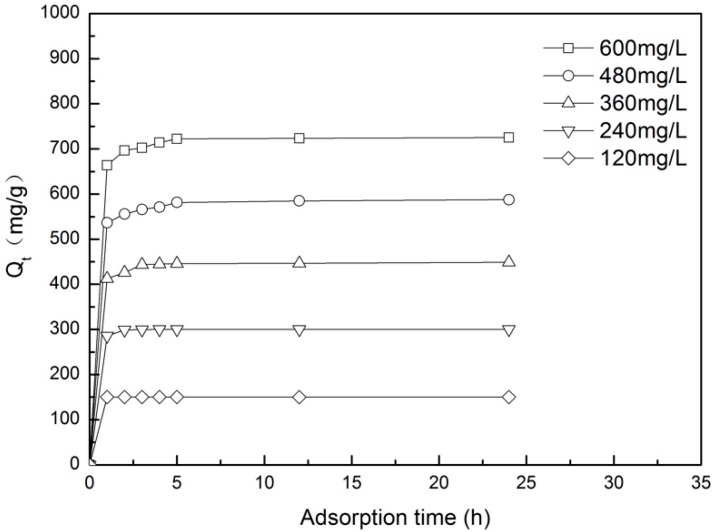
Adsorption kinetic curves of MB with different initial concentrations onto Z-LWACF.

The curve of the adsorption amount of Z-LWACF-6 in MB solution which under the initial concentrations of 120 mg/L, 240 mg/L, 360 mg/L, 480 mg/L, and 600 mg/L over time is shown in Figure 7. It can be seen from the figure that all the adsorption curves of Z-LWACF-6 at different initial concentrations have the same trend. As the initial concentration increased, the equilibrium adsorption capacity of sample increased gradually. It was due to the rise of concentration leading to increase chances of contact between adsorbate molecules and sample surface [34]. When the initial concentration was 120 mg/L~240 mg/L, the adsorption rate of MB which can reach the adsorption equilibrium in a short time was very fast, and the adsorption amount of sample in equilibrium is 150 mg/g~299.75 mg/g. In contrast, the time of adsorption equilibrium was prolonged when the initial concentration is from 360 mg/L to 600 mg/L. However, the adsorption of MB at different initial concentrations all reached the equilibrium state while the adsorption time was 5 h, and the adsorption value of Z-LWACF-6 at equilibrium was 446.12 mg/g to 725.63 mg/g. Besides, with the prolongation of adsorption time, the instantaneous adsorption rate of Z-LWACF-6 was firstly faster and gradually became slower as equilibrium position was approached [35]. This was mainly because Z-LWACF-6 needed to undergo three processes for the adsorption of MB in solution. The first stage was the diffusion and transfer of adsorbate molecules in solution. Secondly, methylene blue molecules entered the pores of Z-LWACF-6 under the action of pore diffusion. Finally, it was adsorbed on the active sites of internal pores [36,37]. As more and more methylene blue molecules were adsorbed, the adsorption rate began to become slow, resulting in a relatively long time required for the adsorption process [38].

### 3.6. Kinetics Analysis

Adsorption kinetic analysis are thoroughly investigated at the condition of 25 °C and initial pH value (6.3–6.5), which were suitable for the adsorption of cationic dyes like MB [20].The adsorption rate and mechanism can be studied by kinetics analysis. The adsorption kinetics can be fitted by pseudo-first-order kinetic equations and pseudo-second-order kinetic equation. These two kinetic models can be described as follows:

(4) $$\ln\left(Q_{e}-Q_{t}\right)=\ln Q_{e}-k_{1}t$$

(5) $$\frac{t}{Q_{t}}=\frac{1}{k_{2}Q_{e}^{2}}+\frac{1}{Q_{e}}t$$

where *Q*_e_ and *Q*_t_ (mg/g) represent the amounts of MB adsorption at equilibrium state and any time (h), respectively. *k*_1_ (h^−1^) is the rate constant of the pseudo-first-order model and *k*_2_ (g·mg^−1^·h^−1^) is the pseudo-second-order rate constant.

**Figure 8:**
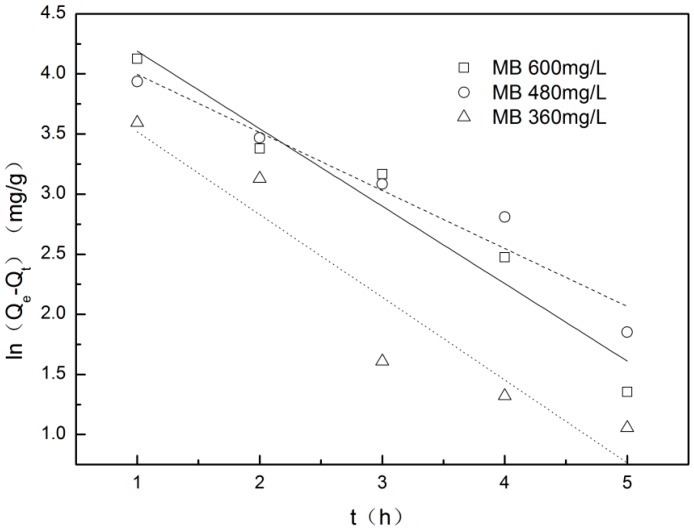
Pseudo-first-order model for MB adsorption onto Z-LWACF.

The pseudo-first-order kinetic model for the adsorption of MB onto Z-LWACF-6 and the equation parameters are shown in Figure 8 and Table 6. Since sample adsorbed MB quickly to reach the maximum adsorption amount at initial concentrations of 120 mg/L and 240 mg/L, only the relevant data of initial concentration of 360 mg/L, 480 mg/L and 600 mg/L have been fitted here. As can be seen from the figure, the data points were highly dispersive and the linear relationship was poor. According to the data in the table, the correlation coefficient *R*^2^ of the pseudo-first-order kinetics of Z-LWACF-6 adsorbing MB at different initial concentrations was 0.9078~0.9505, and the fitting effect was poor. When the initial concentration increased, the theoretical equilibrium adsorption amount increased gradually. It implied that the equilibrium adsorption amount was closely related to the initial concentration of MB. However, the theoretical value and the actual equilibrium adsorption amounts were quite different, indicating that the pseudo-first-order model could not accurately describe the whole process of MB adsorption onto Z-LWACF-6. According to previous literature [39], this would be attributed to the intraparticle mass transfer resistance which was the limiting factor for MB adsorption in pseudo-first-order kinetics studies.

**Figure 9:**
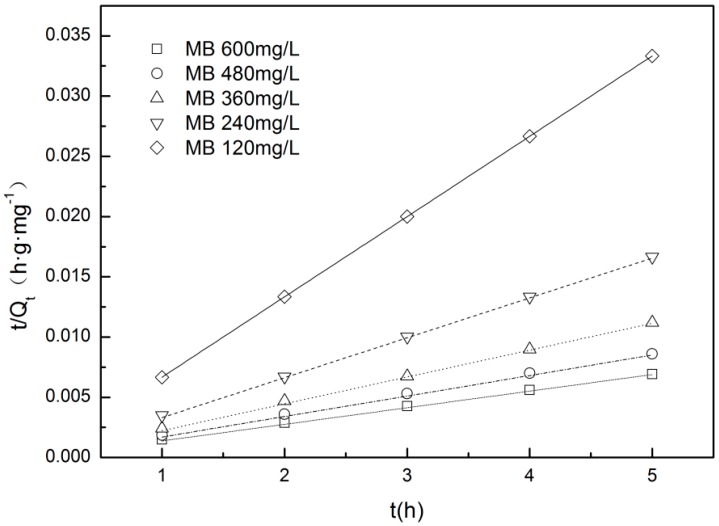
Pseudo-second-order model for MB adsorption onto Z-LWACF.

Figure 9 and Table 7 show the pseudo-secondary kinetic model fitting and equation parameters of MB adsorption onto Z-LWACF-6. It can be seen from Figure 9 that the experimental data at different concentrations have a good linear relationship. From Table 8, with the increase of initial concentration, the adsorption rate constant *k*_2_ gradually decreased from 0.4489 to 0.0098. It was obvious that the theoretical equilibrium adsorption calculated by pseudo-secondary kinetics was close to the experimentally measured equilibrium adsorption at different initial concentrations. In addition, the correlation coefficient of pseudo-secondary dynamics fitting was between 0.9998~1 and the fitting results were better than pseudo-first-order kinetics. This indicated that the pseudo-second-order kinetics was more suitable for describing the process of Z-LWACFs for MB adsorption [40], including external liquid membrane diffusion, surface adsorption and internal diffusion of particles, which can more accurately reflect the adsorption mechanism of MB on Z-LWACFs.

### 3.7. Adsorption Isotherms

Langmuir and Freundlich models were adopted to analysis the isotherm data of MB adsorption onto Z-LWACFs. These two models can be expressed as follows:

(6) $$\frac{C_{e}}{Q_{e}}=\frac{1}{K_{L}Q_{m}}+\frac{C_{e}}{Q_{m}}$$

(7) $$\ln Q_{e}=\ln K_{F}+\frac{1}{n}\ln C_{e}$$

where *K*_L_ (L/mg) is the Langmuir constants involved in adsorption rate, and *Q*_m_ (mg/g) is adsorption amount. *K*_F_ and 1/*n* are Freundlich constant and empirical constant.

**Figure 10:**
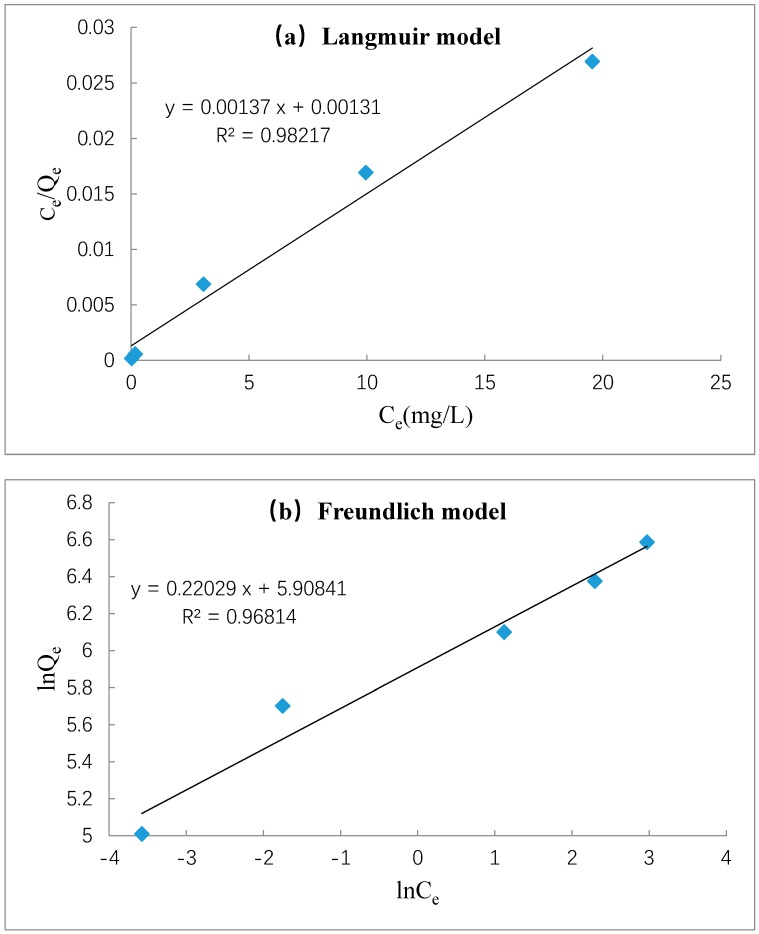
Linear fit of Langmuir model (**a**) and Freundlich model (**b**).

The experimental data were fitted using Equations (6) and (7). The Langmuir and Freundlich isotherms are displayed in Figure 10a,b respectively. The fitting parameters can be calculated from these two isotherms and their value are shown in Table 8. As can be seen from the table, the correlation coefficients *R*^2^ (0.9822) of the Langmuir adsorption isotherms was close to 1 and better than Freundlich model (0.9682), indicating the good application of Langmuir model [41]. Furthermore, the equilibrium adsorption amount determined from the Langmuir model was 729.93 mg/g, which was close to the actual equilibrium adsorption amount. All the results manifested that the Langmuir isotherm model was more suitable for the adsorption of MB onto Z-LWACF, and the surface adsorption form was monolayer adsorption. In addition, the parameter 1/*n* calculated by Equation (7) is frequently used to characterize the adsorption intension. When 1/*n* is in the range of 0.1 to 0.5, it indicates that the adsorption is easy occurred. From the table, the 1/*n* value was 0.22, indicating that MB is easy to be adsorbed by Z-LWACFs.

## 4. Conclusions

The trend of MB adsorption amount of Z-LWACF at different impregnation ratios was consistent with pore structure distribution above 1.5 nm pore size, indicating that larger micropores (1.5 nm to 2 nm) and mesopores played a major role in the MB adsorption by Z-LWACF. The iodine adsorption capacity of Z-LWACF is consistent with the change of pore structure at pore diameters of 0.8 nm to 1.5 nm and 2 nm to 4 nm. Under different initial concentrations, the adsorption amount of Z-LWACF increased rapidly with the increase of adsorption time. With the increase of initial concentration, the equilibrium adsorption capacity of Z-LWACF increased gradually. The fitting results show that the pseudo-second-orde kinetic model is more suitable for describing the adsorption process of Z-LWACF to MB, indicating that the rate of MB adsorption is controlled by chemisorption. The results of adsorption isotherms show that the adsorption of MB by Z-LWACF belongs to monolayer adsorption and Z-LWACF is easy to adsorb MB.

## Figures and Tables

**Table 1:** Parameter of MB solution for standard curve.

MB Concentration (mg/L)	UV Absorbancy
0.24	0.031
0.48	0.054
1.2	0.136
2.4	0.264

**Table 2:** The yield and S_BET_ of ZACF produced by one and two step methods and others ACFs.

Sample	Activating Agent	Activation Temperature (°C)	Activation Time (min)	Yield (%)	BET Surface Area (m^2^/g)
ZACF-700	ZnCl_2_	700	60	53.79	1086
ZACF2-700	ZnCl_2_	700	60	44.37	762
WACF-700 ^a^	Steam	700	60	49.00	784
ACF-700WB ^b^	Steam	700	60	43.45	716
ACF-700 ^c^	Na_2_HPO_4_	700	60	54.44	560
WACFs-700 ^d^	CO_2_	700	40	54.75	490
ACF-1~3 ^e^	KOH	850	60	66.8~35.6	536~1371

^a^: Liu et al. (2012) ^b^: Zhang et al. (2013) ^c^: Wu et al. (2015) ^d^: Li et al. (2013) ^e^: Huang et al. (2016).

**Table 3:** Results of the fits of C1s region of Z-LWACFs prepared at different impregnation ratios.

Sample	Graphite (C–C) (%)	C–O (%)	C=O (%)	–COOH (%)
	BE = 284.7 (eV)	BE = 285.6 (eV)	BE = 286.5 (eV)	BE = 288.3 (eV)
Z-LWACF-3	59.66	19.25	13.77	7.31
Z-LWACF-4	56.13	22.75	12.14	8.98
Z-LWACF-5	54.73	24.25	13.45	7.56
Z-LWACF-6	51.08	22.20	16.07	10.66

**Table 4:** MB adsorption value and pore volume of Z-LWACFs prepared at different impregnation ratios.

Sample	Pore Volume (cm^3^/g)			MB Adsorption Value (mg/g)
	*V*_total_	*V*_micro_	*V*_meso_	
Z-LWACF-3	0.446	0.237	0.191	251
Z-LWACF-4	0.598	0.326	0.251	359
Z-LWACF-5	0.872	0.371	0.439	560
Z-LWACF-6	0.953	0.376	0.566	641

**Table 5:** Iodine adsorption values of Z-LWACFs prepared at different impregnation ratios.

Sample	Pore Volume (cm^3^/g)			Iodine Adsorption Value (mg/g)
	*V*_total_	*V*_micro_	*V*_meso_	
Z-LWACF-3	0.446	0.237	0.191	880
Z-LWACF-4	0.598	0.326	0.251	1005
Z-LWACF-5	0.872	0.371	0.439	1117
Z-LWACF-6	0.953	0.376	0.566	1159

**Table 6:** Kinetic parameters of pseudo-first-order model for MB adsorption onto Z-LWACF.

MB Concentration (mg/L)	*Q*_e_ (mg/g)	*R* ^2^	*Q*_e_ (mg/g)	*k*_2_ (g/(mg·h))
600	725.63	0.9505	123.92	0.6447
480	587.63	0.9499	86.97	0.4827
360	446.12	0.9078	66.32	0.6884
240	299.75	-	-	-
120	150	-	-	-

**Table 7:** Kinetic parameters of pseudo-second-order model for MB adsorption onto Z-LWACF.

MB Concentration (mg/L)	*Q*_e_ (mg/g)	*R* ^2^	*Q*_e_ (mg/g)	*k*_2_ (g/(mg·h))
600	725.63	0.9999	714.28	0.0098
480	587.63	0.9998	588.24	0.0145
360	446.12	0.9998	454.55	0.0161
240	299.75	0.9999	303.03	0.1089
120	150	1.0000	149.25	0.4489

**Table 8:** Fitting parameters of different adsorption isotherm model.

Langmuir Model			Freundlich Model		
*R* ^2^	*Q*_m_ (mg/g)	*K*_L_ (L/mg)	*R* ^2^	1/*n*	*K*_F_ (mg/g)
0.9822	729.93	0.96	0.9681	0.22	361.54

## References

[1] Bedin K.C., Martins A.C., Cazetta A.L., Pezoti O., Almeida V.C. (2016). KOH-activated carbon prepared from sucrose spherical carbon: Adsorption equilibrium, kinetic and thermodynamic studies for Methylene Blue removal. Chem. Eng. J..

[2] Zollinger H. (1991). Hallux rigidus and its treatment. Therapeutische Umschau Revue Thérapeutique.

[3] Li Y., Du Q., Liu T., Liu T., Peng X., Wang J., Sun J., Wang Y., Wu S., Wang Z., Xia Y., Xia L. (2013). Comparative study of methylene blue dye adsorption onto activated carbon, graphene oxide, and carbon nanotubes. Chem. Eng. Res. Des..

[4] Reema R.M., Saravanan P., Kumar M.D., Renganathan S. (2011). Accumulation of Methylene Blue Dye by Growing Lemna minor. Sep. Sci. Technol..

[5] Pathania D., Sharma S., Singh P. (2013). Remova lof methylene blue by adsorption onto activated carbon developed from Ficus carica bast. Arab. J. Chem..

[6] Yusof N., Rana D., Ismail A.F., Matsuura T. (2016). Microstructure of polyacrylonitrile-based activated carbon fibers prepared from solvent-free coagulation process. J. Appl. Res. Technol..

[7] Zhao X., Lai S., Liu H., Gao L. (2009). Preparation and characterization of activated carbon foam from phenolic resin. J. Environ. Sci..

[8] Park S.H., Kim C., Jeong Y.I., Lim D.Y., Lee Y.E., Yang K.S. (2004). Activation behaviors of isotropic pitch-based carbon fibers from electrospinning and meltspinning. Synth. Met..

[9] Saeidi N., Lotfollahi M.N. (2015). Effects of powder activated carbon particle size on adsorption capacity and mechanical properties of the semi activated carbon fiber. Fibers Polym..

[10] Zhang F., Ma X.J. (2013). Effect of Spinning Process on the Absorption Performance of Wooden Activated Carbon Fiber for Methylene Blue. Appl. Mech. Mater..

[11] Ma X., Zhang F., Wei L. (2015). Effect of wood charcoal contents on the adsorption property, structure, and morphology of mesoporous activated carbon fibers derived from wood liquefaction process. J. Mater. Sci..

[12] He L.F., Liu Q.X., Ji T., Gao Q. (2012). Preparation and Characterization of Activated Carbon Fibers from Jute Fibers by Phosphoric Acid Activation. Appl. Mech. Mater..

[13] Liu Q.S., Zheng T., Li N., Wang P., Abulikemu G. (2010). Modification of bamboo-based activated carbon using microwave radiation and its effects on the adsorption of methylene blue. Appl. Surf. Sci..

[14] Sasaki T., Matsumoto A. (2011). Effect of pore structure and particle size of activated carbon on adsorption rate of cigarette smoke constituents. Carbon.

[15] Phan N.H., Rio S., Faur C., Coq L.L., Cloirec P.L., Nguyen T.H. (2006). Production of fibrous activated carbons from natural cellulose (jute, coconut) fibers for water treatment applications. Carbon.

[16] Yu B., Huang Z.H., Wang M.X., Kang F. (2013). Adsorption of benzene and ethanol on activated carbon nanofibers prepared by electrospinning. Adsorption.

[17] Lin C.L., Cheng Y.H., Liu Z.S., Chen J.Y. (2013). Adsorption and oxidation of high concentration toluene with activated carbon fibers. J. Porous Mater..

[18] Rathore R.S., Srivastava D.K., Agarwal A.K., Verma N. (2010). Development of surface functionalized activated carbon fiber for control of NO and particulate matter. J. Hazard. Mater..

[19] Alma M.H., Baştürk M.A., Shiraishi N. (2001). Cocondensation of NaOH-Catalyzed Liquefied Wood Wastes, Phenol, and Formaldehyde for the Production of Resol-Type Adhesives. Ind. Eng. Chen. Res..

[20] Huang Y., Ma E., Zhao G. (2015). Preparation of liquefied wood-based activated carbon fibers by different activation methods for methylene blue adsorption. RSC Adv..

[21] Li D.N., Ma X.J. (2013). Effect of activation technology on wooden activated carbon fiber structure and iodine adsorption property. J. Funct. Mater..

[22] Liu Z.G., Huang Y.X., Zhao G.J. (2016). Preparation and Characterization of Activated Carbon Fibers from Liquefied Wood by ZnCl_2_ activation. Bioresources.

[23] (1999). Test Methods of Wooden Activated Carbon-Determination of Methylene Blue Adsorption.

[24] (1999). Test Methods of Wooden Activated Carbon-Determination of Iodine Number.

[25] Liu W.J., Zhao G.J. (2012). Effect of temperature and time on microstructure and surface functional groups of activated carbon fibers prepared from liquefied wood. Bioresources.

[26] Zhang J., Zhang W. (2013). Preparation and characterization of activated carbon fibers from liquefied poplar bark. Mater. Lett..

[27] Vu T.T., Liu W.J., Zhao G.J. (2015). Pore structure and surface chemistry structure of Na_2_HPO_4_ activated liquefied wood carbon fiber. Acta Mater. Compos. Sci..

[28] Li D.N., Ma X.J. (2013). Preparation and characterization of activated carbon fibers from liquefied wood. Cellulose.

[29] Huang Y., Zhao G. (2016). Preparation and characterization of activated carbon fibers from liquefied wood by KOH activation. Holzforschung.

[30] Ma X., Zhang F., Zhu J., Yu L., Liu X. (2014). Preparation of highly developed mesoporous activated carbon fiber from liquefied wood using wood charcoal as additive and its adsorption of methylene blue from solution. Bioresour. Technol..

[31] Wang S., Zhu Z.H., Coomes A., Haghseresht F., Lu G.Q. (2005). The physical and surface chemical characteristics of activated carbons and the adsorption of methylene blue from wastewater. J. Colloid Interface Sci..

[32] Jin Z., Yan X., Yu Y., Zhao G. (2014). Sustainable activated carbon fibers from liquefied wood with controllable porosity for high-performance supercapacitors. J. Mater. Chem. A.

[33] He F. (2004). Carbon Fiber and Its Application Technology.

[34] Juang R.S., Wu F.C., Tseng R.L. (2000). Mechanism of Adsorption of Dyes and Phenols from Water Using Activated Carbons Prepared from Plum Kernels. J. Colloid Interface Sci..

[35] Auta M., Hameed B.H. (2012). Modified mesoporous clay adsorbent for adsorption isotherm and kinetics of methylene blue. Chem. Eng. J..

[36] Pezoti O., Cazetta A.L., Souza I.P.A.F., Bedin K.C., Martins A.C., Silva T.L., Almeida V.C. (2014). Adsorption studies of methylene blue onto ZnCl_2_-activated carbon produced from buriti shells (*Mauritia flexuosa* L.). J. Ind. Eng. Chem..

[37] Hameed B.H., Din A.T.M., Ahmad A.L. (2007). Adsorption of methylene blue onto bamboo-based activated carbon: Kinetics and equilibrium studies. J. Hazard. Mater..

[38] Senthilkumaar S., Varadarajan P.R., Porkodi K., Subbhuraam C.V. (2005). Adsorption of methylene blue onto jute fiber carbon: kinetics and equilibrium studies. J. Colloid Interface Sci..

[39] Liu Q.X., He L.F., Yang J.H., Ji T., Gao Q. (2012). Adsorption of jute fiber activated carbon on methylene blue and methyl orange solutions. Ind. Text..

[40] Han R., Zhang L., Song C., Zhang M., Zhu H., Zhang L. (2010). Characterization of modified wheat straw, kinetic and equilibrium study about copper ion and methylene blue adsorption in batch mode. Carbohydr. Polym..

[41] Fu J.W., Chen Z.H., Wang M.H., Liu S.J., Zhang J.H., Zhang J.N., Han R.P., Xu Q. (2015). Adsorption of methylene blue by a high-efficiency adsorbent (polydopamine microspheres): Kinetics, isotherm, thermodynamics and mechanism analysis. Chem. Eng. J..

